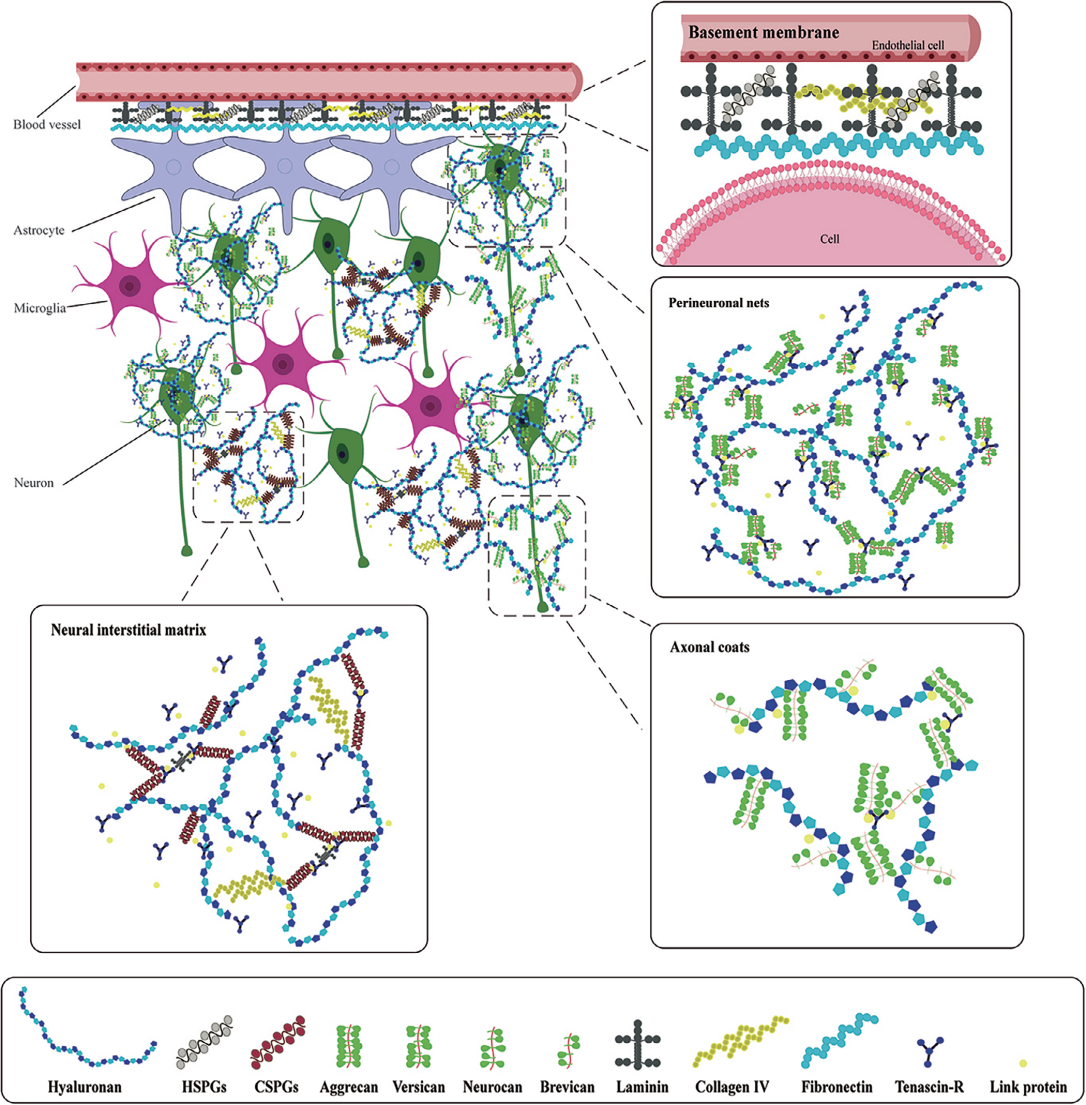# The critical role of extracellular matrix in Alzheimer's disease

**DOI:** 10.1002/alz70855_101712

**Published:** 2025-12-27

**Authors:** Xiaolei Zhu, Zhen Lan, Yun Xu

**Affiliations:** ^1^ Affiliated Drum Tower Hospital of Nanjing University Medical School, Nanjing, China; ^2^ Affiliated Drum Tower Hospital of Nanjing University Medical School, Nanjing, Jiangsu, China

## Abstract

**Background:**

In central nervous system, all components are enveloped by the extracellular matrix (ECM), regulating microenvironmental homeostasis and neuronal development. The condensed cartilage‐like ECM called perineuronal nets (PNNs), mainly surrounding parvalbumin‐expressing (PV+) fast‐firing GABAergic interneurons, are crucial to synaptic plasticity and memory modulation.

**Method:**

PNNs are developed in the vital periods of synaptic maturation and play a critical role in synaptogenesis, synaptic stability, neuroprotection and ionic buffering. In addition, ECM includes basement membranes (BM) and neural interstitial matrix (NIM). All types of ECM are composed of chondroitin sulphate proteoglycans (CSPGs), heparan sulphate proteoglycans (HSPGs), hyaluronan, glycoproteins and other molecules secreted by neurons and glial cells.

**Result:**

In Alzheimer's disease (AD), ECM acts as a physical and chemical barrier to interact with pathological hallmarks such as β‐amyloid (Aβ), hyperphosphorylated tau protein, synaptic alternation and excitatory toxicity. These components of ECM are dynamically changed to modulate neuronal functions and cellular interactions.

**Conclusion:**

We highlight ECM, especially the perineuronal ECM, and its components in AD, and elaborate the potential therapeutic targets in AD.